# High serological and molecular prevalence of *Ehrlichia canis* and other vector-borne pathogens in dogs from Boa Vista Island, Cape Verde

**DOI:** 10.1186/s13071-024-06437-9

**Published:** 2024-09-04

**Authors:** Rocio Checa, Laura Peteiro, Belén Pérez-Hernando, María de la Morena, Lourdes Cano, Pedro López-Suárez, Juan Pedro Barrera, Efrén Estévez-Sánchez, Juliana Sarquis, Blanca Fernández-Cebrián, Ana Montoya, Guadalupe Miró

**Affiliations:** 1 Animal Health Department, Veterinary School, Universidad Complutense de Madrid, Madrid, Spain; 2 Microbiology and Parasitology Department, Faculty of Pharmacy, Universidad Complutense de Madrid, Madrid, Spain; 3 Bios Cabo Verde, Boa Vista, Republic of Cabo Verde

**Keywords:** *Anaplasma platys*, Boa Vista, Dog, *Ehrlichia canis*, *Rhipicephalus sanguineus* s.l.

## Abstract

Despite the high global impacts of canine vector-borne diseases (CVBD) due to their wide distribution and zoonotic potential, the current epidemiological situation of CVBD in many tropical and subtropical regions remains unknown. This study examines the seroprevalence and molecular prevalence of *Ehrlichia canis* and other pathogens causing CVBDs (*Leishmania infantum*, *Dirofilaria immitis, Babesia* spp., *Anaplasma* spp. and *Hepatozoon canis*) in dogs living on the island of Boa Vista (Cape Verde Republic). Blood samples and infesting ticks were taken from 150 dogs across the island (stray, shelter, and pet dogs). Serum samples were tested using a rapid immunochromatographic test (Uranotest^®^ Quattro) that detects antibodies against *E. canis*, *L. infantum*, *Anaplasma* spp. and *D. immitis* antigen. Levels of serum antibodies against *E. canis* were measured using the immunofluorescence antibody test (IFAT). In addition, tick-borne pathogens in blood samples (*Anaplasma* spp., *Babesia* spp., *Hepatozoon* spp., and *Ehrlichia canis*) were detected by microscopy observation and/or PCR plus sequencing. The seroprevalence of *E. canis* was extremely high at 82% (123/150), as revealed by both immunochromatography and IFAT. Most dogs returning a seropositive test result (82.92%; 102/123) had antibody titres > 1:1280 but showed no clinical signs or notable laboratory abnormalities. Of the 123 animals testing seropositive for *E. canis*, 67 (54.47%) also presented antibodies against *Anaplasma* spp*.,* and 13 (10.56%) showed the presence of *Hepatozoon* spp. gamonts in the blood smear. *Ehrlichia canis* infection was detected in 17.1% (25/146) of dogs tested by direct sequencing of polymerase chain reaction (PCR) products. Co-infections were detected in seven of these dogs: four dogs tested PCR-positive for both *E. canis* and *A. platys*, two dogs tested positive for *E. canis* and *Hepatozoon* spp., and one dog tested positive for *E. canis*, *A. platys* and *Hepatozoon* spp. *Rhipicephalus sanguineus* sensu lato was the only tick species found infesting the canine study population. The high prevalence of tick-borne pathogens detected in dogs from Boa Vista Island highlights a need for improved control measures designed to prevent the transmission of these pathogens.

**Graphical Abstract:**

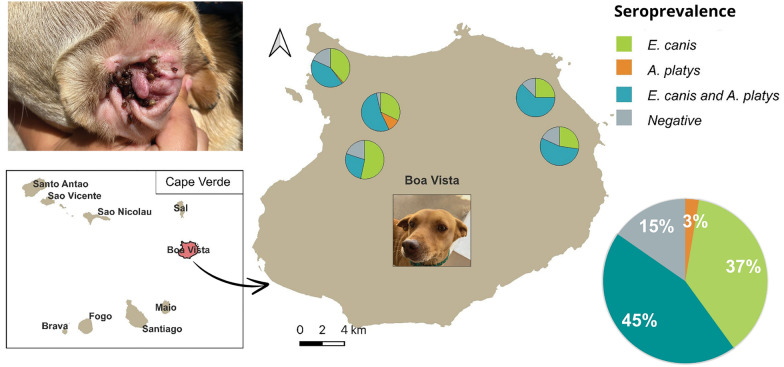

## Background

Canine vector-borne diseases (CVBD) are a complex group of diseases (e.g. ehrlichiosis, anaplasmosis, babesiosis, heartworm or leishmaniosis), some of which have zoonotic potential [1].

The etiological agent of canine monocytic ehrlichiosis (CME) is *Ehrlichia canis*, a Gram-negative intracellular bacterium classified within the group of alpha-proteobacteria, order Rickettsiales, family *Anaplasmataceae*, genus *Ehrlichia* [2]. The main vector responsible for transmitting this emerging rickettsial disease is *Rhipicephalus sanguineus*, commonly known as “brown dog tick”. Canine monocytic ehrlichiosis is a multi-systemic disease with three clinical stages: acute (occurring 8–20 days after tick transmission with non-specific clinical signs), subclinical (dogs appear healthy but can exhibit blood abnormalities) and chronic (severe haemorrhagic and ocular disorders) [3]. The diagnosis of CME should be based on clinical records, laboratory findings and serological and molecular tests [4]. In endemic areas, monitoring seropositive but healthy dogs is essential, as reinfection can occur due to the lack of persistent immunity.

Since the discovery in 1935 of *Ehrlichia canis* in dogs from Algeria, other *Ehrlichia* species have been described to infect dogs, including *Ehrlichia ewingii*, causing granulocytic ehrlichiosis, and *Ehrlichia chaffeensis*, causing human monocytic ehrlichiosis. Both can infect dogs and have been detected in several tick species and other vertebrate hosts in many countries. *Ehrlichia ruminantium* has been detected in healthy dogs and those exhibiting clinical signs using molecular diagnostic tests. *Ehrlichia muris* was identified in a sick dog in northern Minnesota that had tested seronegative for *E. canis* [5].

Canine anaplasmosis is mainly caused by two species, *Anaplasma phagocytophilum*, which mainly infects neutrophils causing canine granulocytic anaplasmosis, and *A. platys,*, which affects platelets causing infectious cyclic thrombocytopenia [4]. *Anaplasma phagocytophilum* is transmitted by ticks of the genus *Ixodes* and is, therefore, more prevalent in regions with a temperate climate. This species of *Anaplasma* can infect a wide variety of animals, including dogs, cats, sheep, goats, cows, deer and humans [6]. The arthropod vector that transmits *A. platys* has not been identified, although the brown dog tick is a major candidate [4]. Co-infection with *E. canis* and *A. platys* is frequent in dogs where *R. sanguineus* s.l. is present. Also common is co-infection with other pathogens transmitted by this tick species, such as *Babesia vogeli* or *Hepatozoon canis* [7, 8].

Canine babesiosis is a severe tick-borne haemoprotozoan disease caused by various *Babesia* species worldwide. In Africa and Europe, three large *Babesia* species (*Babesia canis*, *Babesia rossi* and *Babesia vogeli*) and two small species (*Babesia gibsoni* and *Babesia vulpes*) exist and these have been described as clinically relevant [9]. The distribution of *Babesia* spp. is closely related to their vector’s range; *B. canis* and *B. vulpes* are mainly found in Europe, while *B. vogeli* and *B. gibsoni* are distributed globally, including tropical and subtropical areas. *Babesia rossi* is restricted to Africa and causes severe clinical disease [10].

Canine hepatozoonosis is caused by two species of *Hepatozoon*. *Hepatozoon canis* is a tick-borne protozoan parasite that infects dogs globally. Transmitted mostly by *R. sanguineus* s.l., the parasite resides within the host’s neutrophils and affects haemolymphoid organs, causing systemic illness. Another more pathogenic species, *Hepatozoon americanum*, has described in dogs from the USA [11].

Other significant zoonotic CVBDs that are not transmitted by ticks are leishmaniosis and heartworms. Canine leishmaniosis is detected in the Mediterranean Basin, North Africa, South America, and West Asia. Numerous species of *Leishmania* infect dogs in tropical regions. *Leishmania infantum* is the most widespread and is transmitted by phlebotomine sandflies [12]. Infected dogs may exhibit a wide range of non-specific clinical signs, such as muscular atrophy, lymph node enlargement, skin lesions, and renal and ocular disorders, among others [13]. Thus, outside endemic areas, the diagnosis of this disease can be challenging for veterinarians. Heartworm is caused by *Dirofilaria immitis*, a cosmopolitan filarial worm transmitted by mosquitoes (Culicidae) to various domestic and wild carnivores [14]. It is also sporadically detected in humans in regions where this parasite is endemic in dogs [15]. The disease typically progresses chronically in dogs due to the pathogenic effect of adult worms residing in the pulmonary arteries.

The varied clinical picture presented by CVBD makes their diagnosis extremely complex. Canine monocytic ehrlichiosis and anaplasmosis are globally distributed tick-borne diseases of major concern as they can be life-threatening for dogs if not adequately treated. Canine monocytic ehrlichiosis has been reported on all continents, mostly in tropical and subtropical regions where environmental conditions promote the survival of its vector, *R. sanguineus*. However, the current epidemiological situation of CVBD in many tropical and subtropical regions still needs to be defined. The emergence of CVBD is influenced by globalization, urbanization, and climate change, particularly increased temperatures and altered rainfall patterns. Because of their zoonotic potential, canine vector-borne, particularly tick-borne, diseases are now recognized as emerging global health concerns for humans and animals [16].

The present study was designed to determine the seroprevalence and molecular prevalence of infection by *Ehrlichia canis* and other canine vector-borne pathogens (*Leishmania infantum*, *Dirofilaria immitis, Babesia* spp.*, Anaplasma* spp., and *Hepatozoon canis*) in dogs living on the island of Boa Vista.

## Methods

### Geographical study area

Boa Vista is an island occupying 620 km^2^ that forms part of the Cape Verde archipelago in the Atlantic, off the north-west coast of Africa. It is one of Cape Verde’s easternmost islands (16° 06′ 12″ N; 22° 48′ 13″ O). Its main town is Sal Rei.

Cape Verde has a tropical dry climate influenced by the north-easterly trade winds. Its rainy season typically occurs between July and October, although mean annual precipitation is relatively low. Vegetation tends to be arid, with plants adapting to the scarce water conditions. Climate and vegetation vary among the different islands owing to differences in altitude and topography.

### Study population

**Fig. 1:**
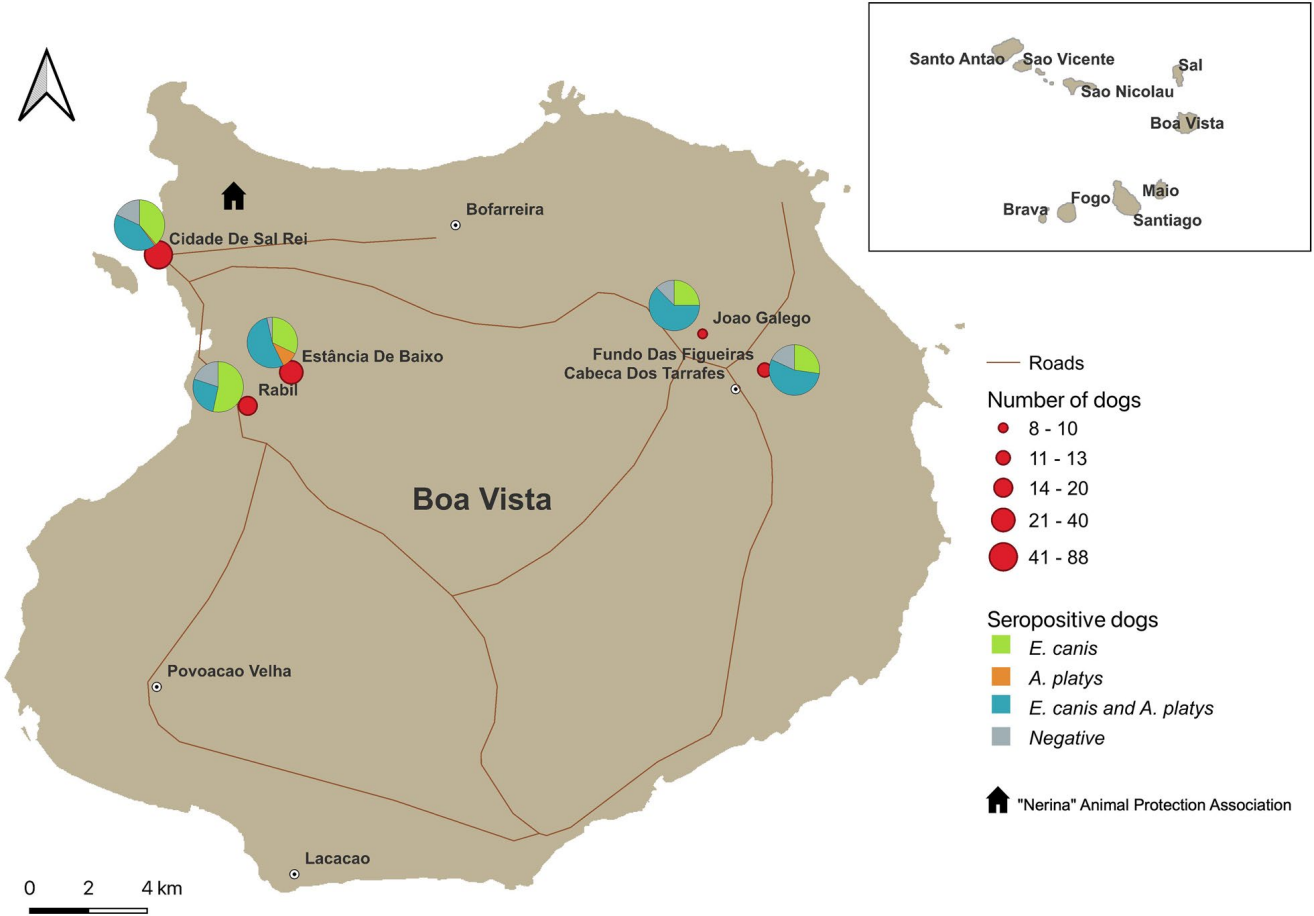
Sampling sites (red dots) and distribution areas of dogs testing seropositive for *Ehrlichia canis* and *Anaplasma platys* infection in Boa Vista. The size of the red dot indicates the number of dog samples analysed per sampling site. Pie charts show the proportions of seropositive dogs by sampling site

This was a cross-sectional study in which 150 dogs from Boa Vista Island were enrolled. Participating dogs were divided into four groups on the basis of their lifestyle: companion dogs (*n* = 23), stray dogs that were homeless yet cared for and fed by a responsible person (*n* = 74), stray dogs with no person responsible for them (*n* = 11), and dogs living at a shelter run by the NERINA Animal Protection Association (*n* = 31) (Fig. 1).

### Samples and data collection

Blood and ticks were collected from dogs in April 2022 during a sterilization programme carried out by NERINA to mitigate overpopulation.

All participating dogs were subjected to a thorough physical exam in which the data—date, location, age, breed, sex, weight, and lifestyle—were collected along with clinical findings.

Three millilitres of blood were drawn by cephalic or jugular venipuncture and immediately transferred to two EDTA tubes (0.5 ml each) and a tube without anticoagulant (2 ml). Serum was then separated by centrifugation. Serum and EDTA-mixed blood samples were initially kept at 4 °C for complete blood counts, blood smears and a rapid immunochromatographic test. Subsequently, all samples were stored at −20 °C until serological and molecular processing in the laboratory.

### Clinicopathological tests

Complete blood counts were performed in 102 of the 150 participating dogs (due to financial restrictions) including red blood cell count (RBC), haematocrit, haemoglobin concentration, mean corpuscular volume (MCV), mean corpuscular haemoglobin concentration (MCHC), mean corpuscular haemoglobin (MCH), leukocyte count (WBC) and platelet count. For this purpose, a Mythic TM 18 Vet haematology analyser (Orphée S.A., Geneva, Switzerland) was used.

### Microscopy observation of stained blood smears

Thin blood smears (*n* = 139) were Diff-Quick stained and examined under a light microscope to detect *Ehrlichia* spp., *Anaplasma* spp., *Hepatozoon* spp. and piroplasm species or microfilariae.

### Immunological diagnosis

Serum samples (*n* = 150) were subjected to a rapid immunochromatographic test (ICT) (Uranotest® Quattro, Spain) to detect antibodies against *E. canis*, *L. infantum*, *Anaplasma* spp., and *D. immitis* antigen. In addition, an immunofluorescence antibody test (IFAT) was used to determine anti-*Ehrlichia canis* serum IgG antibodies using a commercially available antigen kit (Anti-*Ehrlichia* Antibodies, Biosystems, Barcelona, Spain). The IFAT test was performed according to the manufacturer’s instructions. A cut-off of 1:80 was taken to indicate seropositivity. Positive sera were further tested in a serial dilution starting at 1:20.

### Molecular diagnosis

DNA from whole blood was isolated using the QIAamp® DNA mini kit (Qiagen, Germany) following the manufacturer’s instructions. In the last step, DNA was eluted in 200 µl and stored at −20 °C until further use.

Blood DNA samples (*n* = 146) were analysed using different polymerase chain reaction (PCR) methods to detect tick-borne pathogens including piroplasm species, *Ehrlichia canis* and *Anaplasma* spp.

The 146 DNA samples were tested using an *Ehrlichia* genus-specific PCR assay targeting approximately 838 bp of 16S ribosomal DNA (rDNA). This PCR method was performed as described by Peter et al. [17]. Briefly, a final volume of 20 µl reaction mixture was prepared containing 5 µl of genomic DNA, 12.4 µl Master mix (Biotools, Spain) and a 10 µM final concentration of each primer (EHRF and EHRR). The amplification conditions were an initial denaturation step of 95 °C for 5 min, followed by 35 cycles of denaturation at 95 °C for 45 s, annealing at 62 °C for 45 s and extension at 72 °C for 45 s, and a final extension at 72 °C for 7 min.

In addition, a conventional PCR was performed to detect *Anaplasma* spp. only in blood samples from sick dogs with a PCR-positive result for *E. canis* (*n* = 25) or a positive serology result for *Anaplasma* spp. (*n* = 5). A total of 30 DNA samples were tested using an *Anaplasma* genus-specific PCR method following the protocol described by Peter et al. [17], using the primers ANAF and ANAR, which targeted an 424 bp of *Anaplasma* 16S rDNA. The PCR conditions were the same as those described for the *Ehrlichia* genus-specific PCR assay. The amplification conditions were an initial denaturation step of 95 °C for 5 min, followed by 40 cycles of denaturation at 95 °C for 45 s, annealing at 57 °C for 45 s and extension at 72 °C for 45 s, and a final extension at 72 °C for 7 min.

To estimate the incidence of *Babesia* spp. on the island, a piroplasm genus-specific nested PCR was run on 12.6% of randomly selected dog DNA samples (*n* = 19) using primer sets BTF1 and BTF2 F/R, which target the 18S ribosomal RNA (rRNA) gene, as described elsewhere [18].

PCR amplifications were performed in a thermocycler GenAmp^®^ PCR System 2700 (Applied Biosystems, Spain). PCR products (10–15 µl) were run on a 1.5% agarose gel containing SYBR Safe Gel Stain (Invitrogen, USA) and visualized with a dark reader transilluminator.

### Sequencing

All PCR products corresponding to the expected length were sequenced at the Genome Sequencing Service (Universidad Complutense de Madrid, Spain) using an ABI Prism 3730 instrument (Applied Biosystem, Foster City, USA). The DNA sequence chromatogram files obtained were aligned with Chromas software, edited sequences were compared with sequence databases, and significance was determined using the BLAST® programme (https://blast.ncbi.nlm.nih.gov/Blast.cgi).

### Ectoparasite identification

Ectoparasites collected (*n* = 349) from dogs after their clinical examination were placed in individual tubes containing 70% ethanol. Ectoparasites (255 ticks and 94 fleas) were classified to the species level according to morphological features using identification keys [19–22]. In addition, they were sexed and the tick stage (larvae, nymph or adult) was determined.

### Statistical analysis

Statistical tests were performed using the IBM SPSS Statistics package (version 28.0). A descriptive analysis was performed using absolute and relative frequencies for qualitative variables, and the mean, standard deviation and percentiles for quantitative variables. The chi-squared test was used to examine relationships between *E. canis* and *Anaplasma* spp. seropositivity and the remaining categorical variables. The non-parametric Kruskal–Wallis test was used to compare blood counts between infected dogs with *E. canis* and/or *A. platys* (PCR positive), dogs seropositive and PCR negative for *E. canis* and/or *A. platys*, and dogs seronegative and PCR negative for *E. canis* and *A. platys*. Significance was set up at *P* < 0.05.

## Results

### Serology

Of the 150 serum samples screened using the rapid immunochromatographic test (Uranotest® Quattro), 127 (84.7%) returned seropositive results as follows: 56 (37.3%) were seropositive only for *E. canis*, 4 (2.6%) for *Anaplasma* spp. only, and 67 (44.6%) for *E. canis* and *Anaplasma* spp. simultaneously (Fig. 1). However, all tested dog serum samples were negative for *L. infantum* antibodies and *D. immitis* antigen.

The seroprevalence of *E. canis* infection was 82% (123/150) as determined by immunochromatography and IFAT. Anti-*Ehrlichia* IgG antibody titres were 1/80 to 1/5120 as follow: 1/80 (*n* = 4), 1/160 (*n* = 1), 1/320 (*n* = 3), 1/640 (*n* = 3), 1/1280 (*n* = 14), 1/2560 (*n* = 53) and 1/5120 (*n* = 45). Most seropositive dogs with high antibody titres (≥ 1:1280; *n* = 112) showed no clinical signs or notable laboratory abnormalities (64.3%; 77/112).

The epidemiological variables recorded in dogs testing seropositive for *E. canis* and *Anaplasma* spp*.* are shown in Tables 1 and 2, respectively. No significant differences were found in the factors study area, age, size or reproductive status and seropositivity for *E. canis* or *Anaplasma* spp. However, the factor breed did differ significantly among the groups established, and a relationship was found between exposure to *E. canis* infection and mixed breed (*P* = 0.002). According to lifestyle, stray and shelter dogs showed the highest seroprevalences, 85.3% (99/116) for *E. canis* and 52.6% for *Anaplasma* spp*.* (61/116), varying significantly with respect to companion animals which had a seropositivity of 65.2% (15/23) for *E. canis* (*P* = 0.02) and 21.7% (5/23) for *A. platys* (*P* = 0.007).

**Table 1:** Epidemiological variables recorded in dogs testing seropositive (*n* = 123) or seronegative (*n* = 27) for *E. canis*

Variable	*n*	Positive (%)	Negative (%)	*χ*^2^	*df*	*P*-value
Study area						
Sal-Rei	88	71 (80.7)	17 (19.3)	0.570	4	0.966
Rabil	15	12 (80.0)	3 (20.0)			
Estancia de Baixo	28	24 (85.7)	4 (14.3)			
Joao Galego	8	7 (87.5)	1 (12.5)			
Fundo das Figueiras	11	9 (81.8)	2 (18.2)			
Breed						
Crossbreed	97	79 (81.4)	18 (18.6)	9.559	1	0.002*
Pure breed	12	5 (41.7)	7 (58.3)			
Unknown	41	39 (95.1)	2 (4.8)			
Age (years)						
< 2	33	22 (66.7)	11 (33.3)	5.596	2	0.061
2–4	56	48 (85.7)	8 (14.3)			
> 4	29	25 (86.2)	4 (13.8)			
Unknown	32	28 (87.5)	4 (12.5)			
Body weight (kg)						
< 10	30	22 (73.3)	8 (26.7)	2.227	2	0.328
10–25	100	85 (85.9)	15 (15.0)			
> 25	7	6 (85.7)	1 (14.3)			
Unknown	13	10 (76.9)	3 (23.1)			
Sex						
Female	70	57 (81.4)	13 (18.6)	0.138	1	0.711
Male	68	57 (83.8)	11 (16.2)			
Unknown	12	9 (75.0)	3 (25.0)			
Reproductive status						
Sterilized	93	79 (84.9)	14 (15.1)	0.943	1	0.332
Non-sterilized	36	28 (77.8)	8 (22.2)			
Unknown	21	16 (76.2)	5 (23.8)			
Habitat						
Pet	23	15 (65.2)	8 (34.8)	7.134	3	0.068
Owner/stray	74	65 (87.8)	9 (12.2)			
Shelter dog	31	24 (77.4)	7 (22.6)			
Stray dog	11	10 (90.1)	1 (9.1)			
Unknown	11	9 (81.8)	2 (18.2)			
Lifestyle						
Companion animal	23	15 (65.2)	8 (34.8)	5.27	2	0.02*
Stray/shelter dog	116	99 (85.3)	17(14.6)			
Unknown	11	9 (81.8)	2 (18.2)			
Outdoors						
Yes	85	69 (81.2)	16 (18.8)	0.006	1	0.936
No	33	27 (81.8)	6 (18.2)			
Unknown	32	27 (84.4)	5 (15.6)			
Living with other animal species						
Yes	90	72 (80.0)	18 (20.0)	0.133	1	0.716
No	35	29 (82.9)	6 (17.1)			
Unknown	25	22 (88.0)	3 (12.0)			
Clinical status						
Sick	27	18 (66.7)	9 (33.3)	2.988	1	0.084
Healthy	95	78 (82.1)	17 (17.9)			
Unknown	28	27 (96.4)	1 (3.6)			
Ticks						
Yes	45	40 (88.9)	5 (11.1)	3.647	1	0.056
No	53	39 (73.6)	14 (26.4)			
Unknown	52	44 (84.6)	8 (15.4)			

^*^Significant differences observed

**Table 2:** Epidemiological variables recorded in dogs testing seropositive (*n* = 71) or seronegative (*n* = 79) for *Anaplasma* spp.

Variable	*n*	Positive (%)	Negative (%)	*χ*^2^	*df*	*P* value
Study area						
Sal-Rei	88	38 (43.2)	50 (56.8)	7.37	4	0.11
Rabil	15	4 (26.7)	11 (73.3)			
Estancia de Baixo	28	18 (64.3)	10 (35.7)			
Joao Galego	8	5 (62.5)	3 (37.5)			
Fundo das Figueiras	11	6 (54.5)	5 (45.5)			
Breed						
Cross-breed	97	47 (48.5)	50 (51.5)	0.98	1	0.32
Pure breed	12	4 (33.3)	8 (66.7)			
Unknown	41	20 (48.8)	21 (51.2)			
Age (years)						
< 2	33	13 (39.9)	20 (60.6)	1.45	2	0.48
2–4	56	29 (51.8)	27 (48.2)			
> 4	29	15 (51.7)	14 (48.3)			
Unknown	32	14 (43.8)	18 (56.3)			
Body weight (kg)						
< 10	30	13 (43.3)	17 (56.7)	0.82	2	0.66
10–25	100	52 (52)	48 (48)			
> 25	7	3 (42.9)	4 (57.1)			
Unknown	13	3 (23.1)	10 (76.9)			
Sex						
Female	70	35 (50)	35 (50)	0	1	1
Male	68	34 (50)	34 (50)			
Unknown	12	2 (16.7)	10 (83.3)			
Reproductive status						
Sterilized	93	45 (48.4)	48 (51.6)	0.2	1	0.65
Non-sterilized	36	19 (52.8)	17 (47.2)			
Unknown	21	7 (33.3)	14 (66.7)			
Habitat						
Pet	23	5 (21.7)	18 (78.3)	13.19	3	0.004*
Owner/stray	74	45 (60.8)	29 (39.2)			
Shelter dog	31	11 (35.5)	20 (64.5)			
Stray dog	11	5 (45.5)	6 (54,5)			
Unknown	11	5 (45.5)	6 (54.5)			
Lifestyle						
Companion animal	23	5 (21.7)	18 (78.3)	7.32	2	0.007*
Stray/shelter dog	116	61 (52.6)	55 (47.4)			
Unknown	11	5 (45.5)	6 (54.5)			
Outdoors						
Yes	85	41 (48.2)	44 (51.8)	0.32	1	0.57
No	33	14 (42.4)	19 (57.6)			
Unknown	32	16 (50)	16 (50)			
Living with other animal species						
Yes	90	41 (45.6)	49 (54.4)	0.76	1	0.38
No	35	19 (54.3)	16 (45.7)			
Unknown	25	11 (44)	14 (56)			
Clinical status						
Sick	27	11 (40.7)	16 (59.3)	0.26	1	0.60
Healthy	95	44 (46.3)	51 (53.7)			
Unknown	28	16 (57.1)	12 (42.9)			
Ticks						
Yes	45	26 (57.8)	19 (42.2)	3.26	1	0.19
No	53	21 (39.6)	32 (60.4)			
Unknown	52	24 (46.2)	28 (53.8)			

^*^Significant differences observed

### Molecular diagnosis

PCR was able to detect *Ehrlichia* spp. DNA in 17.1% of the blood samples tested (25/146 dogs). Subsequent sequencing of all PCR-positive samples served to identify only the species *E. canis*.

The 25 dogs testing PCR-positive for *E. canis* showed high anti-*E. canis* antibodies as determined by IFAT (between 1/1280 and 1/5120). Additionally, five of these dogs (5/25) scored PCR positive for *A. platys* (Table 3). Further, among the sick dogs with positive serology results for *Anaplasma* spp. but negative results for *E. canis* by PCR (*n* = 5), two tested positive for *A. platys*. *Babesia* spp. were undetected by PCR in all dogs tested.

**Table 3:** Results obtained for PCR tests (*Ehrlichia* spp. and *Anaplasma* spp.) and microscopy observations of blood smears according to the serological status of the dogs

Serology result	*N*	PCR and blood smear results	*n*–%
*E. canis*	56	*E. canis*	9–16.1
		*Hepatozoon* sp.	4–7.1
		Negatives	43–76.8
*E. canis* and *A. platys*	67	*E. canis*	9–13.4
		*E. canis* and *A. platys*	4–5.9
		*Hepatozoon* sp.	6–8.9
		*E. canis* and *Hepatozoon* sp.	2–2.9
		*E. canis* and *A. platys* and *Hepatozoon* sp.	1–1.5
		Negatives	45–67.2
*A. platys*	4	*A. platys*	2–50
		Negatives	2–50
Negative	23	*Hepatozoon* sp.	3–1.3
		Negatives	20–86.9

### Microscopy observation of blood smears

Of the 150 blood samples collected, 139 were suitable for cytological examination (11 blood smears were non-assessable). Of these, 7 (5%; 7/139) showed *E. canis* morulae, a finding subsequently confirmed by PCR and sequencing. In 16 (11.5%; 16/139), *Hepatozoon* spp. gamonts were observed. However, no piroplasm merozoites or microfilariae were detected in any blood smear.

### Co-infections

Seven dogs were simultaneously infected by more than one pathogen detected by PCR and/or blood smear microscopy examination: four dogs tested positive for both *E. canis* and *A. platys*, two dogs tested positive for both *E. canis* and *Hepatozoon* spp., and one dog tested positive for *E. canis*, *A. platys* and *Hepatozoon* spp. (Table 3).

### Clinical signs and clinicopathological findings in infected seropositive dogs

Of the 150 dogs enrolled, 27 showed some clinical signs (18%) as follows: pale mucous membranes (*n* = 5), cutaneous lesions (*n* = 17), ocular lesions (*n* = 3), otitis (*n* = 1) and weight loss (*n* = 1). Complete blood counts were obtained in 102 dogs. Of these, laboratory abnormalities were detected in 37 (36.3%). The main haematological findings were thrombocytopenia (*n* = 28) and anaemia (*n* = 10). Other laboratory abnormalities such as leukocytosis (*n* = 4) or leukopenia (*n* = 1) were detected much less frequently.

All dogs showing laboratory abnormalities were found to be seropositive or infected by at least one pathogen. A total of 11 dogs were infected by *E. canis*, 2 were co-infected by *E. canis* and *A. platys*, and 24 dogs were seropositive (14 for *E. canis* only and 10 for *E. canis* and *Anaplasma* spp.).

Of 123 dogs found seropositive for *E. canis*, 25 returned a positive result by PCR while 98 were negative by PCR. A total of 9 of these 98 seropositive dogs (PCR negative for *E. canis*) showed clinical signs (9.2%): 1 with pale mucous membranes, 7 with cutaneous lesions and 1 with ocular lesions. In addition, blood counts were obtained in 74 of these seropositive dogs. Laboratory abnormalities, mainly thrombocytopenia (24.3%), anaemia and thrombocytopenia (2.7%), and anaemia (4%), were detected in 24 dogs out of the 74 (32.4%). Of the 25 dogs testing PCR positive for *E. canis*, blood count information was obtained in 13 (remaining dogs did not have blood counts). In 93% (12/13), laboratory abnormalities were found, thrombocytopenia with or without anaemia (38% and 39%, respectively) being the most frequent.

Clinicopathological findings in 16 *E. canis*- and/or *A. platys*-infected dogs, 74 dogs seropositive for *E. canis* and/or *A. platys* (PCR-negative), and 12 dogs seronegative and PCR negative are provided in Table 4. Mean haemoglobin concentrations, red blood cell and platelet counts, haematocrit, and MCV and MCHC values were lower in the infected group than in the seropositive and negative groups (*P* < 0.01). However, no differences were found in blood counts between the groups of seropositive and negative dogs (Table 4).

**Table 4:** Clinicopathological findings in *E. canis*- and/or *A. platys*-infected dogs (PCR positive) compared with *E. canis* and/or *A. platys* seropositive (ICT or IFAT positive and PCR negative) and seronegative (ICT or IFAT and PCR negative) dogs

Blood parameter	Reference interval	Group	*n*	Mean	SD	Percentile		*P* value
						25th	75th	
White blood cells (WBC)	6.4–15.9 × 10^3^/μl	Infected^a^	16	12.26	5.38	7.9	16.30	0.402
		Seropositive^b^	74	11.61	3.17	9.60	13.20	
		Negative^c^	12	12.35	1.76	11.20	13.90	
Red blood cells (RBC)	5.6–8 × 10^6^/μl	Infected^a^	16	5.65	1.26	4.60	6.76	< 0.001
		Seropositive^b^	74	7.40	1.12	6.85	8.15	
		Negative	12	7.82	0.99	7.23	8.53	
Haemoglobin (HGB)	13.3–19.2 g/dl	Infected^a^	16	12.24	3.03	9.35	14.80	< 0.001
		Seropositive^b^	74	17.18	3.08	15.50	19.30	
		Negative^c^	12	18.42	3.19	17.20	20.75	
Haematocrit (HCT)	36–54%	Infected^a^	16	36.82	7.64	30.00	42.85	< 0.001
		Seropositive^b^	74	48.26	7.14	45.10	52.70	
		Negative^c^	12	50.18	7.91	47.05	55.75	
Mean corpuscular volume (MCV)	60–75 fl	Infected^a^	16	65.48	4.13	61.85	68.15	0.422
		Seropositive^b^	74	65.35	3.15	63.40	67.10	
		Negative^c^	12	63.97	3.83	63.25	66.10	
Mean corpuscular haemoglobin (MCH)	21–27 g/dl	Infected^a^	16	21.62	1.61	20.35	22.70	0.003
		Seropositive^b^	74	23.15	1.67	22.30	24.20	
		Negative	12	23.44	1.84	32.30	24.40	
Mean corpuscular haemoglobin concentration (MCHC)	34–38 g/dl	Infected^a^	16	33.04	1.88	31.60	33.80	< 0.001
		Seropositive^b^	74	35.44	2.17	34.20	37.10	
		Negative^c^	12	36.60	1.06	36.25	37.40	
Platelets (PLT)	186–547 × 10^3^/μl	Infected^a^	16	148.88	62.34	100.00	194.00	< 0.001
		Seropositive^b^	74	279.97	149.79	185.00	348.00	
		Negative^c^	12	333.75	128.24	246.00	402.00	

SD, standard deviation

^a^Infected: ICT or IFAT (+) and PCR (+)

^b^Seropositive: ICT or IFAT (+) and PCR (−)

^c^Negative: ICT or IFAT (−) and PCR (−)

### Ectoparasite identification

A total of 26 animals were found to have, at least, an ectoparasite infestation during physical examination and parasites were collected for subsequent identification in the laboratory. In total, 349 ectoparasites were identified morphologically, of which 73.07% (255/349) were ticks and 26.93% (94/349) were fleas. The number of ectoparasites collected from the same individual ranged from 1 to 75 for ticks, and 1 to 73 for fleas.

The only tick species found was *Rhipicephalus sanguineus* s.l. According to stage, these were classified as follows: 110 females (43.14%), 124 males (48.63%), 20 nymphs (7.84%), and one larva (0.39%).

The taxonomic classification of *Rhipicephalus,* was based on the hexagonal shape of the basis capituli and the anal groove positioned posterior to the anus. Once classified to the genus level, species identification relied on examining specific parts of the ticks, such as the spiracles. *Rhipicephalus sanguineus* is characterized by spiracle plates with narrow tails, which are less wide than the adjacent festoon. In females of *R. sanguineus*, the separation of porose areas is broad and palp pedicels are short. In males, the shape of the adanal plates is narrow and trapezoidal, the posterior grooves of the scutum are distinct and the caudal appendage is broad in fed males [21, 22].

The 94 fleas collected were identified as *Ctenocephalides canis* (4.26%; 4/94), *Ctenocephalides felis* (5.32%; 5/94) and *Echidnophaga gallinacea* (90.42%; 85/94).

For the taxonomic classification of fleas, we first checked for the presence of genal and pronotal combs. If both combs were present, the head and genal comb morphology were examined. A head that was twice as long as it was wide, and a genal comb with spines I and II of the same length corresponded to *Ctenocephalides felis;* whilst a head of equal length and width, with spine I of the genal comb shorter than spine II, corresponded to *Ctenocephalides canis*. In the absence of combs, the thorax and head were carefully examined. *Echidnophaga gallinacea* has a contracted thorax, and the anterior margin of its head was angled [20].

## Discussion

Our results indicate the presence of three tick-borne pathogens (*E. canis*, *A. platys* and *Hepatozoon* sp.) in dogs from Boa Vista, each with a different prevalence. *Ehrlichia canis* was detected with a frequency of 82% by both ICT and IFAT, and with one of 17.1% by PCR. This study is the first to examine the seroprevalence of this pathogen and highlights the existence of a hyperendemic area of canine ehrlichiosis. The second most prevalent pathogen was *A. platys*, with an overall seroprevalence of 47.2% (44.6% together with *E. canis* and 2.6% alone). This pathogen was PCR detected simultaneously with *E. canis* in five dogs. *Hepatozoon* sp. was also detected in dogs from the island at a prevalence of 11.5% (16/139) and found co-infecting three dogs with *E. canis*. Co-infections are common as different pathogens are transmitted by the same arthropod vector through the tick’s bite or via their ingestion [23]. This makes it difficult to interpret the role of these pathogens in the clinical and pathological manifestations of the animals examined.

Various studies have confirmed the presence of *H. canis*, *A. platys* and *E. canis* on the different islands of Cape Verde. These studies reveal significant differences in CVBD prevalences depending on the study area. On Maio Island, PCR-confirmed prevalences of 3.3% for *E. canis,* 35.9% for *H. canis* and 34.6% for *A. platys* were reported [8], while in Praia, prevalences found for these pathogens were 26.2%, 63.8% and 7.7%, respectively [24].

The signs of CME are non-specific and difficult to assess. Moreover, because of coinfections, some conditions may be worsened or masked. The clinical status of the animals could not be related to the seroprevalence of *E. canis*, as this infection only shows measurable clinical signs during early or severe disease stages. This, along with the mostly high antibody titres obtained > 1:1280 (82.92%; 102/123), indicates that most of the dog population examined had had contact with the agent and was in a sub-acute disease phase. At this stage, dogs show no clinical signs, but do have high serological titres which are maintained over time due to the prolonged duration of *E. canis* antibodies or possible reinfections owing to a lack of persistent and effective immunity towards the pathogen [25]. While in some animals, haematological findings of thrombocytopenia with or without anaemia were detected together with clinical signs compatible with CME (mucosal pallor), the remaining participants showed no findings suggestive of active disease.

The different groups established for our epidemiological analysis of variables that could affect the risk of *E. canis* and *Anaplasma* spp. infection failed to vary in terms of these factors. The lack of a strong link found here between seroprevalence and the origin of the dogs suggests that the prevalence of infection is homogeneous throughout the island, probably reflecting the extension of the *R. sanguineus* vector [26]. Only the factors dog breed and lifestyle were found significantly associated with seroprevalence (*P* < 0.05), with higher rates observed in the mongrel group and stray or shelter dogs. This may be because most dogs living and bred on the island are not pure breeds and being so prevalent, CME will inevitably mostly affect the largest population group. In addition, we must consider that most pure-bred dogs had their owners, similar to companion animals, who could provide individualised care such as tick prophylaxis, which the street animals did not have.

In our microscopy analysis of blood smears, *E. canis* morulae were detected in 7 out of 139 samples and PCR revealed a higher active infection rate (25/146). This proportion of positive blood smears could be higher in cases of acute disease when there are more circulating organisms in infected cells. Microscopy also revealed the presence of co-infections with *Hepatozoon* sp., which was not unexpected considering that this protozoan is transmitted by ingesting the tick *R. sanguineus*, which is regarded as its definitive invertebrate host. Furthermore, other non-vectorial routes, such as the vertical transmission, can be implicated in the spread of *H. canis* in dogs [27]. The presence of *H. canis* was previously described in Cape Verde with its smear identification in 4 of the 57 animals examined [28]. In our study, *A. platys* was also identified by PCR in seven dogs, which is transmitted by the same vector and mentioned in the studies of Lauzi et al. [8] and Götsch et al. [24].

Finally, through morphological identification of ectoparasites collected from dogs using morphological keys, we found that the only tick species on dogs in Boa Vista was *R. sanguineus* s.l. Indeed, this is the only hard tick species found in Cape Verde in recent studies [8, 24], although other species such as *Amblyomma variegatum*, *Margaropus decoloratus (Syn. R. decoloratus)* and *Hyalomma* sp. had been previously reported [26]. The higher percentage of males (48.63%) than females (43.14%) of *R. sanguineus* s.l. in hosts may be attributed to tick males remaining on the host after mating while females detach for oviposition [16]. The habits of street dogs may explain the presence of fleas on these animals. The finding of *C. felis* and *E. gallinacea* is understandable, considering that some animals cohabited with species such as cats, chickens and horses. Although their role as vectors is not very important in domestic animals, they may be involved in transmitting *Dipylidium caninum*, *Acanthocheilonema reconditum*, *Bartonella* spp. and *Rickettsia * spp. Therefore, while they mainly cause flea allergy dermatitis in dogs via their bite (haematophagous), for the prevention and control of CVBD we should still consider their vector role.

The data emerging from this study will help design measures to prevent the risk of transmission of these pathogens between dogs and humans who live on or travel to this island. Some limitations of our study include a need for knowledge of the animals’ history to make a more accurate diagnosis of how the different parasites affect Cape Verdean individuals. Also, difficulties in handling and transporting samples made it difficult to perform a comprehensive analysis of all blood samples.

Knowing the prevalence of tick-borne diseases is important not only to improve current knowledge, but also to help prevent their spread and thereby maintain the health of animal and human populations under a One Health approach [29].

## Conclusions

The seroprevalence of *Ehrlichia canis* in dogs on the island of Boa Vista (Cape Verde) was found to be extremely high. Our results indicate that most dogs had subclinical or chronic infections with no clinical signs or laboratory abnormalities. However, with high antibody titres, which could mean frequent reinfections, these dogs could act as important carriers of the infection. Moreover, as dogs showing clinicopathological abnormalities were infected mostly by *E. canis,*, this pathogen should be considered the most relevant in this geographical area. Further, *Rhipicephalus sanguineus* s.l. was here consolidated as the main vector responsible for the transmission of *Ehrlichia canis* and potentially of *Anaplasma platys* on the island, thus allowing for co-infections with other tick-borne pathogens such as *Hepatozoon* spp.

## Data Availability

All data generated or analysed during this study are included in this published article. Representative sequences were submitted to the GenBank database under the accession numbers: PP940137, PP941105, PP941124, PP941775-PP941778 and PP952009.
